# Clock gene expression and locomotor activity predict death in the last days of life in *Drosophila melanogaster*

**DOI:** 10.1038/s41598-018-30323-x

**Published:** 2018-08-09

**Authors:** Jia Zhao, Guy Robert Warman, James Frederick Cheeseman

**Affiliations:** Department of Anaesthesiology, School of Medicine, the University of Auckland, Park Road, Grafton, Auckland, 1023 New Zealand

## Abstract

The importance of the circadian clock for the regulation of behaviour and physiology, and the molecular control of these rhythms by a set of clock genes are well defined. The circadian clock deteriorates with advancing age but the mechanism underlying is unclear. Here we recorded the expression of two key clock genes in young, middle-aged and old *Drosophila* using transgenic luciferase lines reporting *period* and *timeless in vivo*. We report a novel marker of imminent death in the expression of TIMELESS. In the days immediately preceding death TIMELESS expression increased to at least 150% of previous acrophase values (88.0% of n = 217) and lost circadian rhythmicity, which predicted death equally well in flies of different ages and under light and temperature cycles. We suggest this transient aberrant clock-gene expression is central to the mechanism of the disturbance in circadian behaviour before death (82.7% of n = 342). We also find that PERIOD expression in central-clock neurons remained robust with age, however PERIOD and TIMELESS in peripheral clocks showed a reduction in both expression level and rhythmicity. In conclusion, as flies age the molecular clock gradually declines at the peripheral level but continues to function at the central until days before death.

## Introduction

Aging is the intrinsic and inevitable process of functional deterioration which ultimately leads to death^1^. The circadian clock is the endogenous pacemaker that generates the daily rhythms among most organisms. This clock is affected by time, showing progressive disturbance with advancing age^2^. This effect of aging on the circadian clock is highly conserved among humans and other animals^3^. In humans, the changes in circadian output signals associated with aging include the reduction of the amplitude and earlier timing of circadian rhythms, both evident in body temperature and sleep-wake cycles^4–6^. Mammalian studies on aging demonstrate shifts in the phase of rhythms and weakening of rhythmicity reported in body temperature and locomotor activity patterns^2,7^. Similar to humans and mammals, *Drosophila* show a lengthened free-running period and decline in the overall rest-activity rhythm strength and sleep consolidation with increasing age^8,9^. In addition, *Drosophila* provide a robust and powerful way to investigate clock changes across life time because of their short lifespan (50–80 days)^10^.

It is interesting that clock-controlled behavioural changes before death have also been observed. For instance, aging flies in their last days of life have been found to exhibit arrhythmia^11^. And in laboratory mammals, impairment of several parameters of circadian rhythms, such as body temperature and locomotor activity, is considered a marker for imminent death^12^. Several mammalian studies have shown a marked decrease in the amount and amplitude of activity as well as a loss of ability to maintain entrainment under light-dark (LD) cycles as the animals approach death. Some animals lose their circadian rhythm completely^13–15^. With general acceptance that decline of the circadian clock is a reliable manifestation of aging, it is not hard to imagine that collapse of the clock could be a potential indicator of death. Nonetheless, there has been very little investigation into the mechanism of this phenomenon prior to death.

As well as the decrease in circadian output rhythms^16^, there is some evidence for age-related changes in circadian organization at other levels. The circadian system is composed of multiple circadian oscillators, controlled by a central pacemaker in the suprachiasmatic nucleus (SCN) in mammals^17^ or a cluster of approximate 150 clock-gene expressing neurons in the brain in *Drosophila* (dorsal lateral neurons (LN_d_), large ventral lateral neurons (l-LN_v_), lateral posterior neurons (LPN), small ventral lateral neurons (s-LN_v_), and dorsal neurons 1–3 (DN_1–3_)^18^). There are oscillators in other regions of the brain and peripheral tissues, referred to as peripheral clocks^19^. Currently, whether aging is associated with defects in the central clock, or weakening of the synchronization of peripheral clocks, or both, remains to be seen.

The molecular oscillators within both the central clock and peripheral clocks are driven by the transcription-translation feedback loops of several clock genes, including *period* and *timeless* (*per* and *tim*) in *Drosophila*^20–22^. How the mechanism of the molecular clock and its gene properties change during aging is less well known and the existing evidence is conflicting^10,23^. It has been reported that in the central clock neurons *per* is robustly expressed in aging flies^11^, but this finding has been challenged by a study showing *per* reduction in central clock with age^9^. Studies in aging mammals also showed controversial results with either normal or reduced expression of various clock genes^2,6,7^.

In this study we aim to investigate how the molecular clock, in the brain and in the peripheral tissues, changes intrinsically with increasing age and before death under constant conditions. We also examine whether strong entrainment cues improve age-related functional decline of the clock. By using transgenic luciferase lines, we are able to measure real-time expression of clock genes products PER and TIM in *Drosophila* in three age cohorts (10-, 30- and 50-day-old) for an extended period of time *in vivo*. We use both *8.0-luc* and *XLG-luc* lines to measure PER expression. *8.0-luc* specifically reflects PER expression in subsets of the central-clock dorsal neuronal clusters (DN_1–3_) and LN_d_^22,24^. *XLG-luc* reflects PER expression in all known *per*-expressing cells throughout the body, including central and peripheral tissues^22^. *XLG-luc* is mainly indicative of peripheral clocks because of the small number of central clock neurons compared to thousands of clock cells in the rest of the body. A similar rule applies to *tim-luc*, which therefore reflects TIM expression in peripheral clocks.

## Results

### Age-related changes of PER in central and peripheral clocks and TIM in peripheral clocks

**Figure 1:**
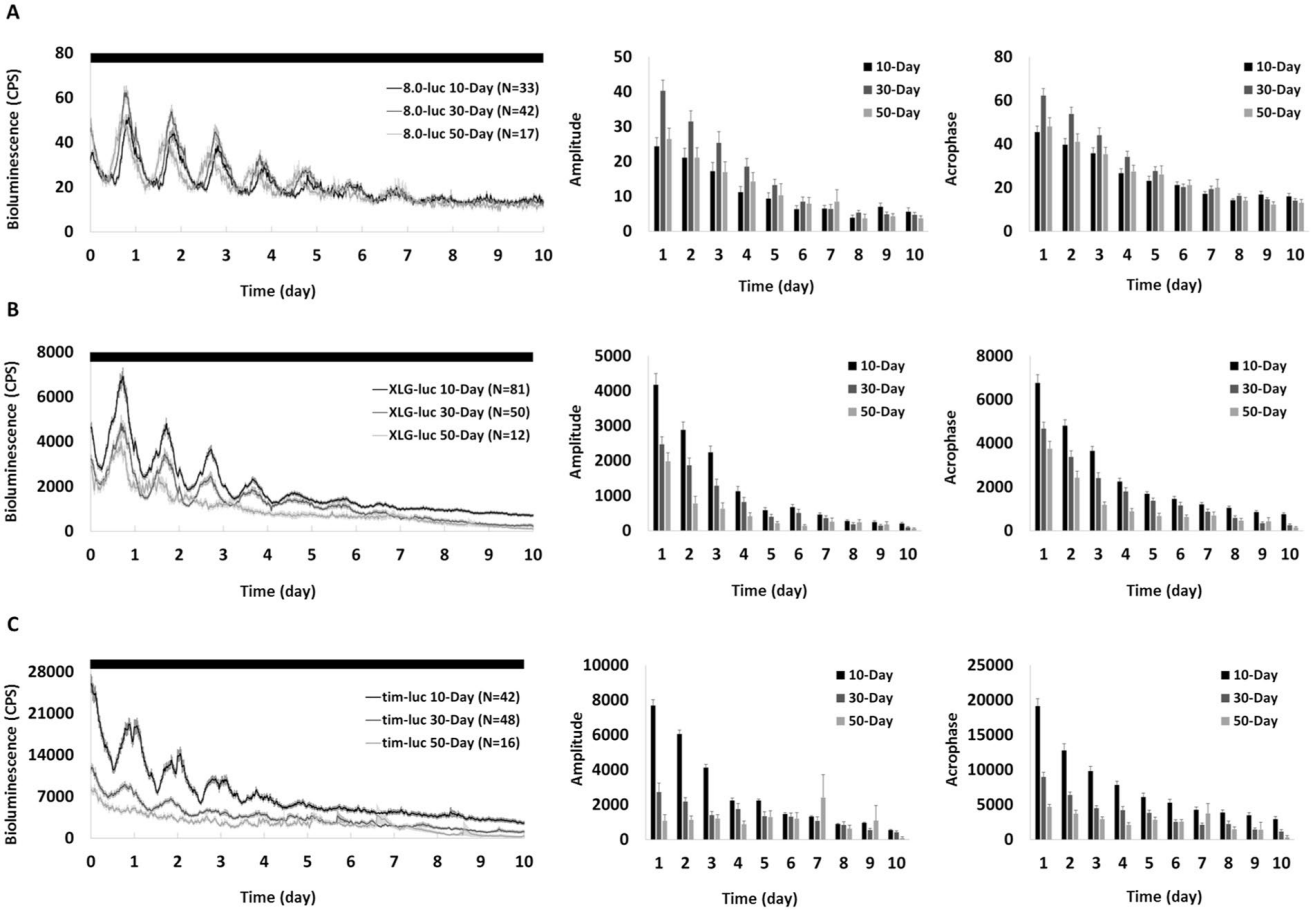
Mean bioluminescence time-series data from young (10-day), middle-aged (30-day) and old (50-day) flies in DD. Bioluminescence counts per second (CPS) is plotted as average of raw counts at 30-minute intervals. Data are shown as mean ± SEM with error bars at each time point. 10-day cohort equals black lines and columns, 30-day cohort equals mid-grey lines and columns, and 50-day cohort equals light-grey lines and columns. (**A**) *8.0-luc* (reporting PER in the dorsal neurons, central clock). The 30-day cohort shows higher PER amplitude and acrophase than 10-day and 50-day (p < 0.001 in all), and there is no difference between 10-day and 50-day (p = 1.00 in both). (**B**) *XLG-luc* (reporting PER in the whole animal, peripheral clocks). The 30-day and 50-day cohort show lower PER amplitude and acrophase compared to 10-day (p < 0.001 in all), 50-day is lower than 30-day (p = 0.02 in amplitude and p = 0.001 in acrophase). (**C**) *tim-luc* (reporting TIM in the whole animal, peripheral clocks). The 30-day and 50-day cohort show lower TIM amplitude and acrophase compared to 10-day (p < 0.001 in all), and 50-day is lower than 30-day in acrophase (p < 0.001) but not in amplitude (p = 0.70).

We analysed the amplitude and acrophase of bioluminescence signal of each day to show the rhythmicity and level of clock-gene expression, respectively. In constant darkness (DD) the rhythmic PER expression at central level in *8.0-luc* middle-aged and old flies was comparable to that in the young flies with robust oscillations (Fig. 1A), showing no decrease in both rhythmicity and expression level. In contrast, both amplitude and acrophase of PER at peripheral level in *XLG-luc* (Fig. 1B) and TIM in *tim-luc* (Fig. 1C) middle-aged and old flies were significantly lower than corresponding young ones, showing age-dependent decline in both rhythmicity and expression level. Despite that the consumption of luciferin (substrate) led to the decay of signal within each age group, by comparing the signal from different age groups at the same time point, the effect of aging on clock-gene expression was still evident.

**Figure 2:**
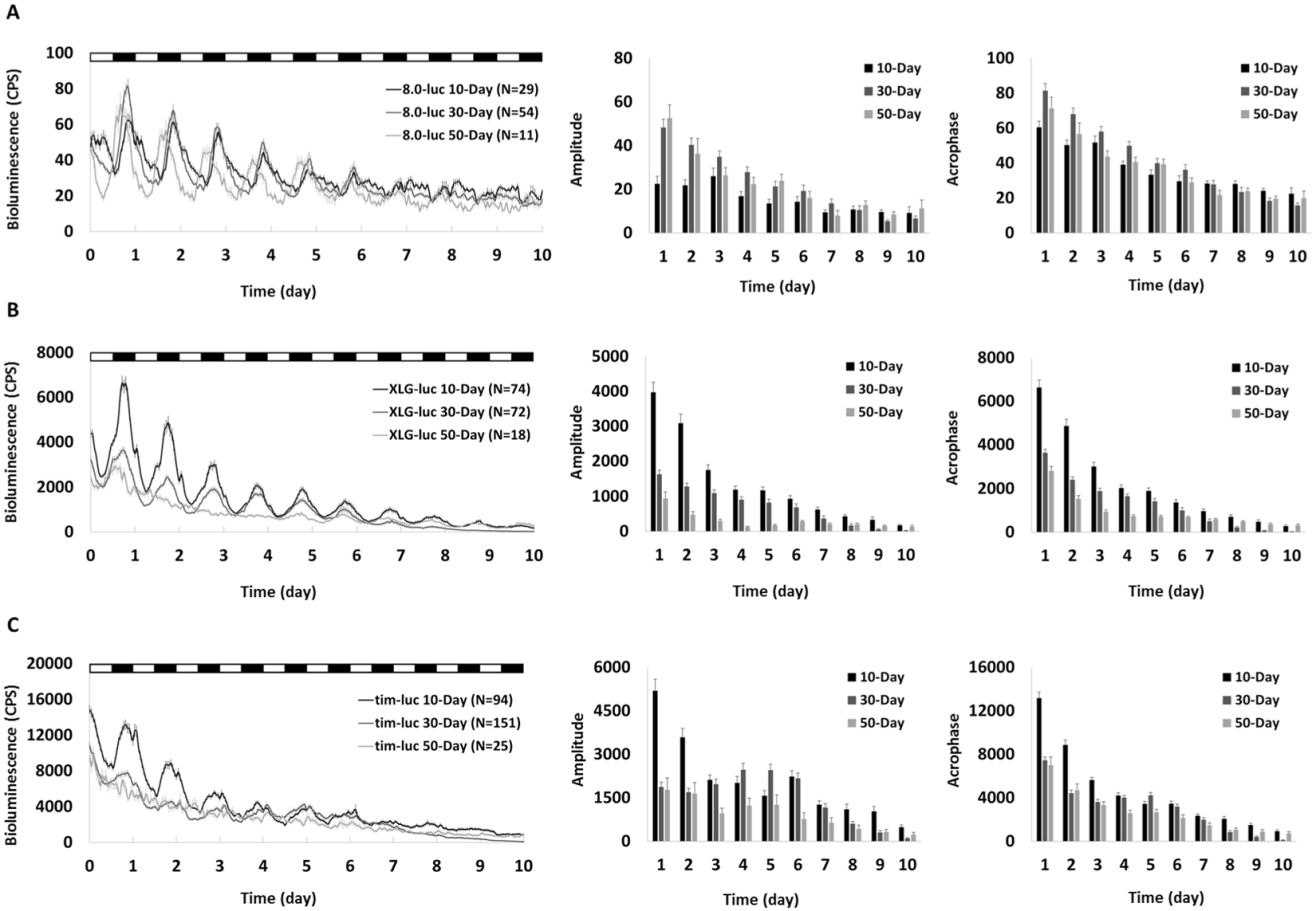
Mean bioluminescence time-series data from young (10-day), middle-aged (30-day) and old (50-day) flies in LD cycles (white bars represent lights on and black bars indicate lights off). Bioluminescence CPS is plotted as average of raw counts at 60-minute intervals. Data are shown as mean ± SEM with error bars at each time point. 10-day cohort equals black lines and columns, 30-day cohort equals mid-grey lines and columns, and 50-day cohort equals light-grey lines and columns. (**A**) The *8.0-luc* 30-day and 50-day cohorts show higher PER amplitude than 10-day (p < 0.001 and p = 0.001, respectively). There is no difference between 30-day and 50-day (p = 1.00). 30-day cohort shows higher PER acrophase than 10-day and 50-day (p < 0.001 and p = 0.02, respectively), and there is no difference between 10-day and 50-day (p = 1.00). (**B**) The *XLG-luc* 30-day and 50-day cohorts show lower PER amplitude and acrophase compared to 10-day (p < 0.001 in all), 50-day is lower than 30-day (p < 0.001 in amplitude and p = 0.001 in acrophase). (**C**) The *tim-luc* 30-day and 50-day cohorts show lower TIM amplitude and acrophase compared to 10-day (p < 0.001 in all), 50-day is lower than 30-day in amplitude (p < 0.001) but not in acrophase (p = 0.11).

**Figure 3:**
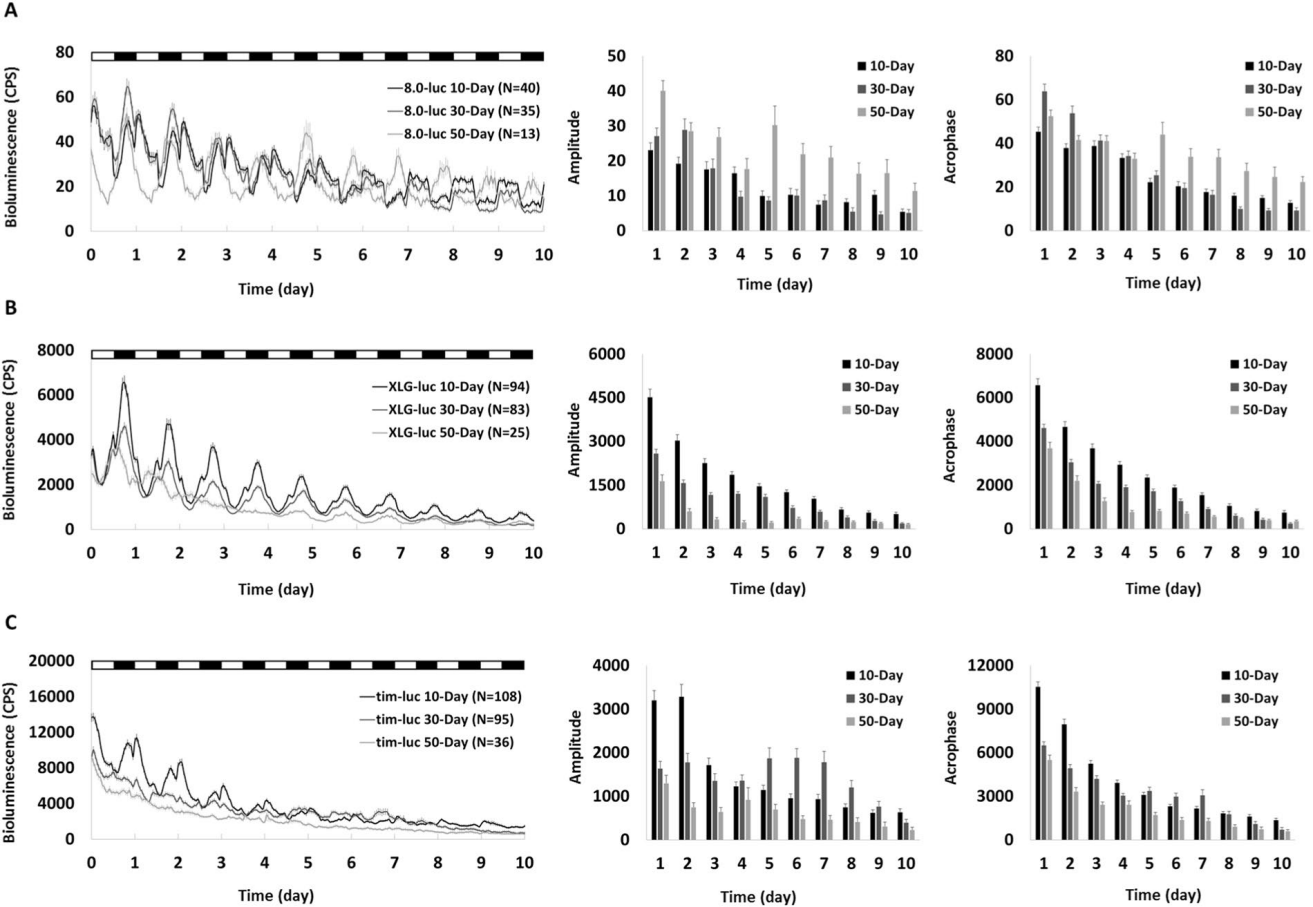
Mean bioluminescence time-series data from young (10-day), middle-aged (30-day) and old (50-day) flies in LD/temperature cycles (lights on at 25 °C and lights off at 21 °C). Bioluminescence CPS is plotted as average of raw counts at 60-minute intervals. Data are shown as mean ± SEM with error bars at each time point. 10-day cohort equals black lines and columns, 30-day cohort equals mid-grey lines and columns, and 50-day cohort equals light-grey lines and columns. (**A**) The *8.0-luc* 50-day cohort shows higher PER amplitude and acrophase than 10-day and 30-day (p < 0.001 in all). 30-day is higher than 10-day in acrophase (p = 0.03) but not in amplitude (p = 1.00). (**B**) The *XLG-luc* 30-day and 50-day cohorts show lower PER amplitude and acrophase compared to 10-day (p < 0.001 in all), 50-day is lower than 30-day (p < 0.001 in both). (**C**) The *tim-luc* 50-day cohort shows lower TIM amplitude and acrophase compared to 10-day and 30-day (p < 0.001 in all), 30-day group is lower than 10-day in acrophase (p < 0.001) but not in amplitude (p = 1.00).

In order to test the influence of entrainment cues on this age-related effect, flies were kept under regular LD cycles with constant temperature and LD coupled with temperature cycles respectively. In LD and LD/temperature cycles the rhythmic PER expressions at central level in *8.0-luc* middle-aged and old flies were comparable to that in the young flies with robust oscillations (Figs 2 and 3A). Similar to that shown in DD, both amplitude and acrophase of PER expression at peripheral level in *XLG-luc* (Fig. 2B) and TIM expression at peripheral level in *tim-luc* (Fig. 2C) middle-aged and old flies were significantly lower than corresponding young ones in LD. Both amplitude and acrophase of PER expression in *XLG-luc* (Fig. 3B) and TIM expression in *tim-luc* (Fig. 3C) old flies were significantly lower than corresponding middle-aged and young ones in LD/temperature cycles.

### Molecular marker of imminent death in both PER and TIM in peripheral clocks

**Figure 4:**
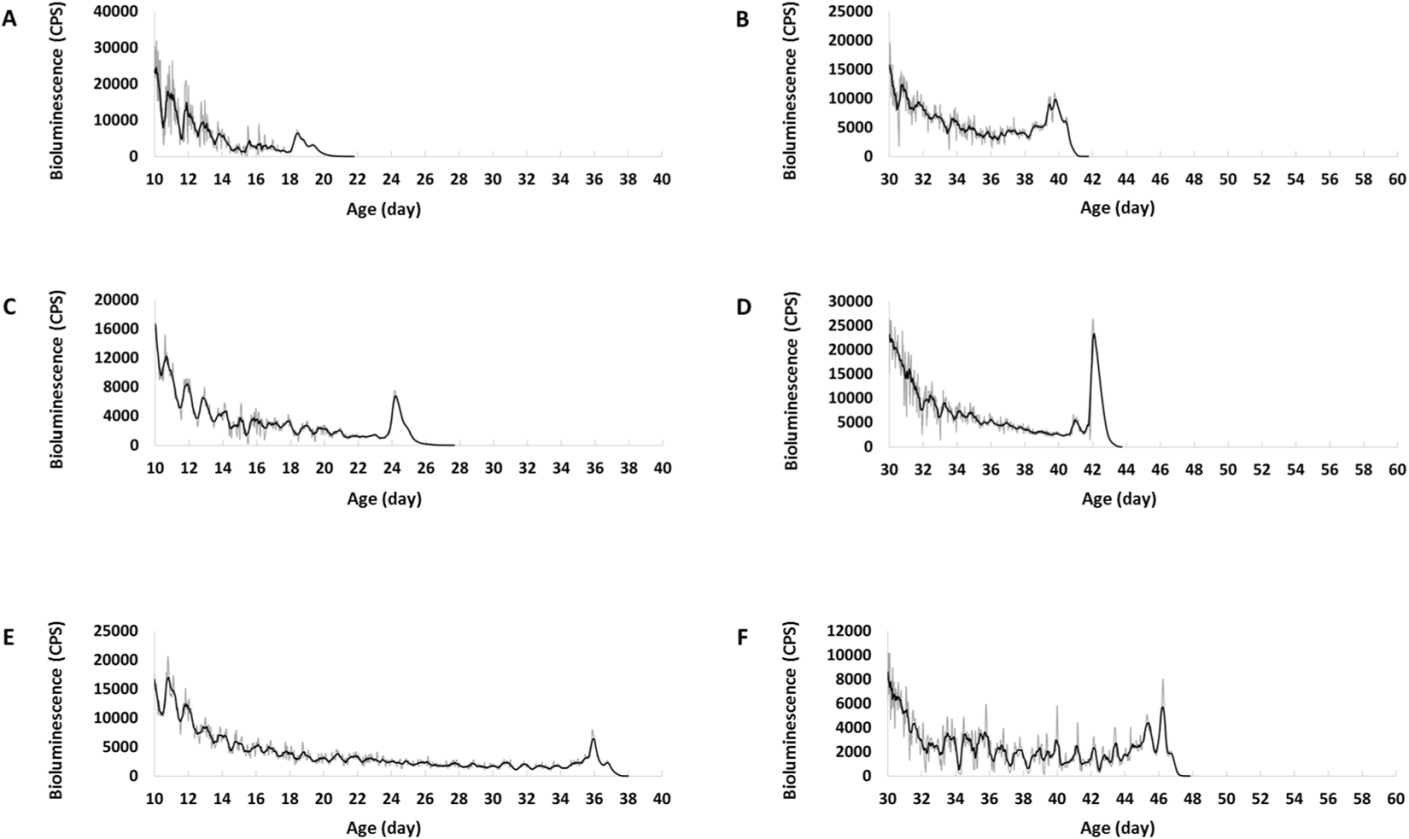
Representative bioluminescence time-series data of *tim-luc* flies showing aberrant TIM signal at the last days of life. X-axis indicates the age of each fly. Bioluminescence CPS is plotted as raw counts (grey lines) and as a 6-hour moving average (black lines). (**A**) A 10-day-old and (**B**) a 30-day-old fly in DD display a rhythmic but descending TIM signal until several days before their deaths at 22 days and 42 days, increases of TIM lasted for 87 hours and 78 hours are observed, respectively. (**C**) A 10-day-old and (**D**) a 30-day-old fly in LD cycles show increases of TIM lasted for 91 hours and 75 hours before their deaths at 27 days and 44 days, respectively. (**E**) A 10-day-old and (**F**) a 30-day-old fly in LD/temperature cycles show increases of TIM lasted for 91 hours and 77 hours before their deaths at 38 days and 48 days, respectively.

**Figure 5:**
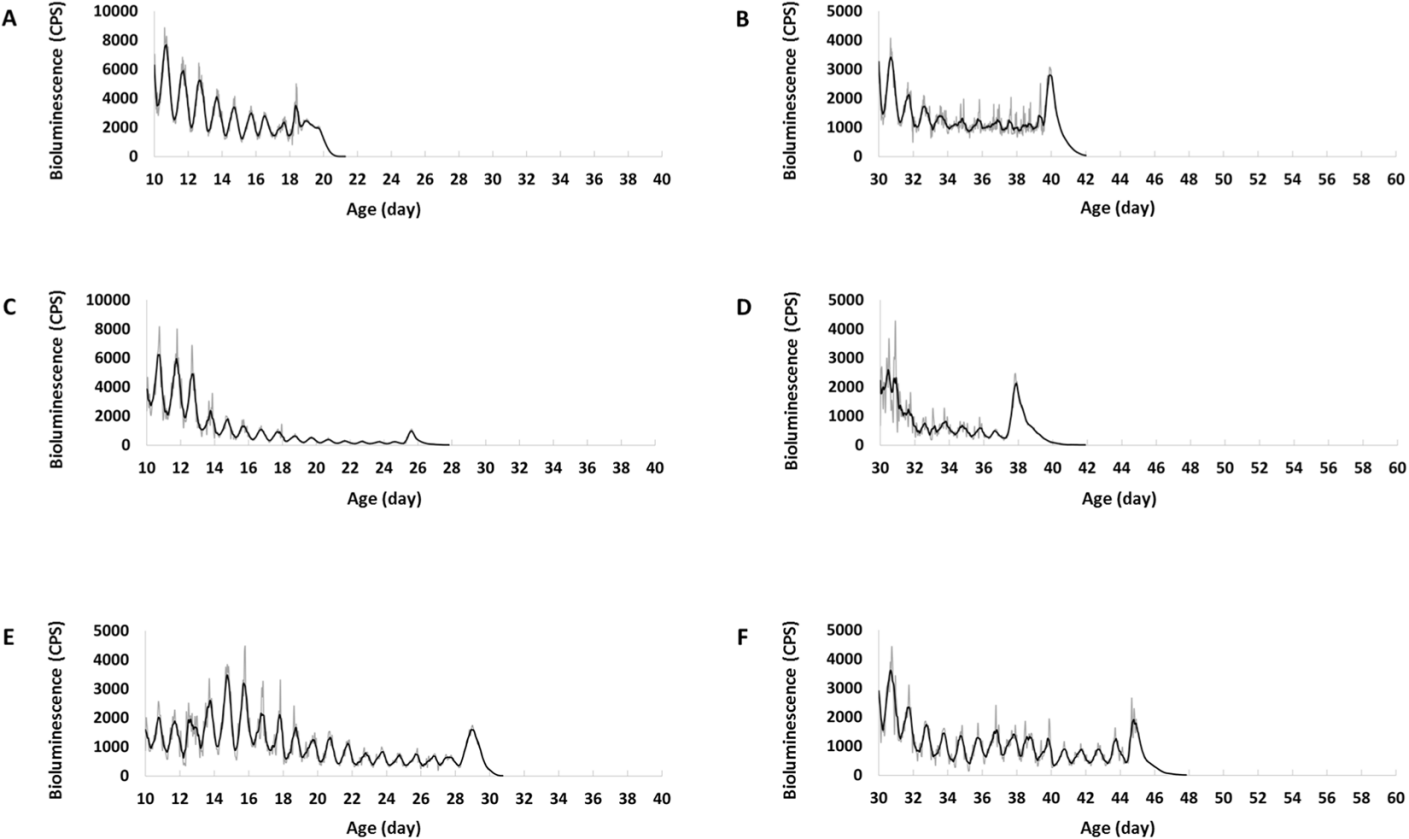
Representative bioluminescence time-series data of *XLG-luc* flies showing aberrant PER signal at the last days of life. X-axis indicates the age of each fly. Bioluminescence CPS is plotted as raw counts (grey lines) and as a 6-hour moving average (black lines). (**A**) A 10-day-old and (**B**) a 30-day-old fly in DD display a rhythmic but descending PER signal until several days before their deaths at 21 days and 42 days, increases of PER lasted for 67 hours and 74 hours are observed, respectively. (**C**) A 10-day-old and (**D**) a 30-day-old fly in LD cycles show increases of PER lasted for 64 hours and 98 hours before their deaths at 28 days and 41 days, respectively. (**E**) A 10-day-old and (**F**) a 30-day-old fly in LD/temperature cycles show increases of PER lasted for 56 hours and 106 hours before their deaths at 31 days and 48 days, respectively.

We found an unexpected increase of TIM signal in *tim-luc* flies (Fig. 4) and PER signal in *XLG-luc* (Fig. 5) at the peripheral level which lasted for several days before eventually vanishing and the animals died. The increase of signal was defined as at least 50% higher than the acrophase of the preceding cycle. Prior to death, 87.8% (n = 82) *tim-luc* flies reared in DD showed an increase in signal over a period of 86.2 ± 2.2 hours, 93.5% (n = 62) in LD cycles showed 98.4 ± 3.4 hours, and 83.6% (n = 73) in LD/temperature cycles showed 90.0 ± 3.0 hours. The age of death of these flies ranged from 20 to 60 days. Prior to death, 34.2% (n = 79) *XLG-luc* flies reared in DD showed an increase in signal over a period of 84.3 ± 5.2 hours, 54.3% (n = 46) in LD cycles showed 81.4 ± 3.8 hours, and 56.3% (n = 48) in LD/temperature cycles showed 81.3 ± 4.6 hours. The age of death of these flies ranged from 20 to 50 days.

**Figure 6:**
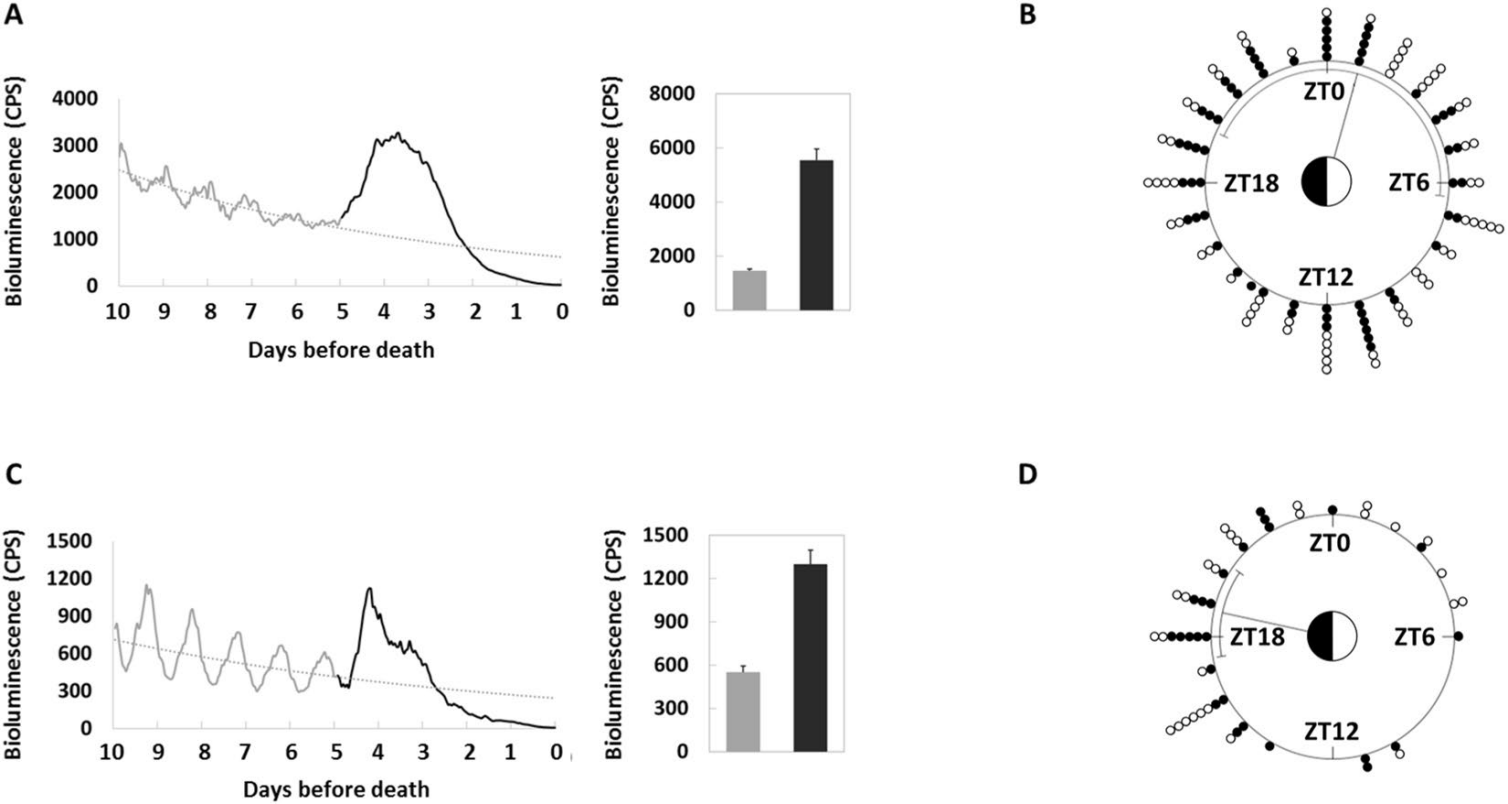
Mean bioluminescence time-series data from the last ten days of life in flies showing elevated signals before death in LD and LD/temperature cycles. X-axis indicates the days before death. Black solid line is the average signal from the last five-days of life, and grey solid line is from the last six to ten days of life with an exponential trendline shown as grey dash line. (**A**) *tim-luc* flies (n = 119) show circadian rhythms of TIM expression from day 10 to day 6 before death with a descending trend. The aberrant signal from the last five days (average height value shown in black column) breaks the circadian pattern and shows a remarkable increase (p < 0.001, compared to the average acrophase from the preceding cycle shown in grey column). (**C**) *XLG-luc* flies (n = 52) show circadian rhythms of PER expression from day 10 to day 6 before death with a descending trend. The aberrant signal from the last five days follows the circadian pattern and shows a remarkable increase (p < 0.001). The circular distributions of the elevated signals before death in a 24-hour clock face. Black solid dots around clock face indicate individual flies in LD cycles and hollow dots in LD/temperature cycles. ZT (where ZT0 is lights on and ZT12 is lights off) is labelled. (**B**) *tim-luc* flies show random distribution in LD (n = 58, p = 0.37) and in LD/temperature (n = 61, p = 0.44). (**D**) *XLG-luc* flies show significant concentrated distribution with mean phase pointing at ZT18 in LD (n = 25, p < 0.001) and at ZT20 in LD/temperature (n = 27, p = 0.007).

In order to confirm that the signal increase occurred before death, we tested how long the bioluminescence signal would last after death under the same condition in a number of flies. Decapitated *tim-luc* flies showed a 11.4 ± 1.6 hour after-death signal (n = 40) and *XLG-luc* flies showed 7.8 ± 1.0 hours (n = 41). In summary, immediately preceding death most *tim-luc* flies (88.0%, n = 217) demonstrated an increase in TIM signal that lasted for 91.2 ± 1.7 hours on average independent of age or environment. At its height this elevated TIM signal was 3.8 times as high as the previous acrophase on average (Fig. 6A), and not concentrated at any particular Zeitgeber time (ZT) calculated by Rayleigh Test (Fig. 6B). In contrast this increase in TIM was not reflected in PER as not as many flies in *XLG-luc* (45.7%, n = 173) showed an increase of 82.3 ± 2.6 hours on average before death. At its height this elevated PER signal was 2.4 times as high as the previous acrophase on average (Fig. 6C), and concentrated with mean phase around ZT18 to ZT20, similar to previous cycles (Fig. 6D).

### Downstream behavioural changes of imminent death

**Figure 7:**
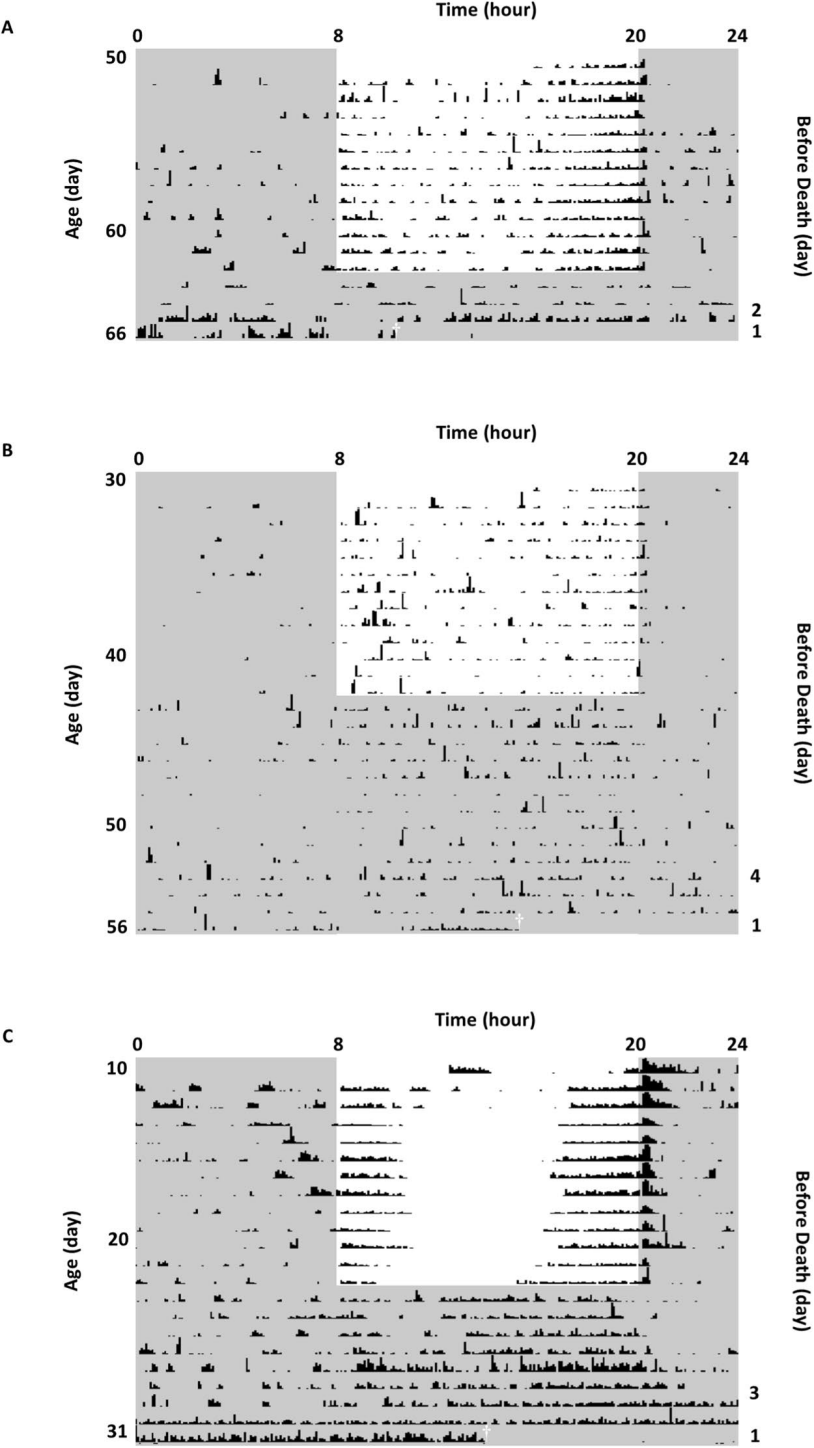
Locomotor activity of representative wild type flies showing arrhythmic behaviour before death. Actogram during a period of LD 12:12 followed by a free running rhythms in DD (the white area marks the light phase, the grey shaded area marks the dark phases). Left y-axis indicates the age of each fly and right y-axis indicates the days before death. Dagger is used as label of death. Well-entrained flies of different ages (**A**) died at 66-day-old, showing a 2-day arrhythmic activity with a FFT value of 0.0001 before death (0.049 in LD and 0.011 in DD), (**B**) died at 56-day-old, showing a 4-day arrhythmic activity with a FFT value of 0.008 before death (0.021 in LD and 0.017 in DD), and (**C**) died at 31-day-old, showing a 3-day arrhythmic activity with a FFT value of 0.0006 before death (0.046 in LD and 0.099 in DD).

Locomotor activity changed in the last few days before death, in accordance to the aberrant PER and TIM expression during a similar period of time. 82.7% of well entrained flies (n = 342) at different ages lost the circadian pattern of behavioural rhythms and became arrhythmic over a period of 3.7 ± 0.1 days before death (Fig. 7). Arrhythmic locomotor activity was observed in 82.9% in flies which started to be entrained and recorded from 10-day-old (n = 146), 85.1% in flies from 30-day-old (n = 148), and 75.0% in flies from 50-day-old (n = 48), showing no significant difference by Chi-square test (p = 0.27). The age of death of these flies ranged from 20 to 70 days. 24-hour relative fast Fourier transform (FFT) values was used as a measure of circadian rhythm strength and FFT value <0.01 was considered arrhythmic. Flies showed a FFT value of 0.050 ± 0.002 under LD entrainment and 0.039 ± 0.002 under following free-running in DD, indicating the existence of circadian rhythms of locomotor activity. FFT value dropped to 0.007 ± 0.0003 (p < 0.001) in the final days preceding death, showing behavioural arrhythmicity.

## Discussion

In our investigation of the effect of aging on the endogenous clock we found a reduction in both amplitude and acrophase of PER and TIM expression rhythms throughout the body in *Drosophila* with advancing age (see Fig. 1). We suggest this reduction in clock gene production and expression rhythmicity indicates the decline of circadian clock function. It has been reported that this reduction in expression as well as rhythmicity is very likely to be the main cause for the changes in the circadian pattern of activity in aging flies^25^. This theory is further supported by gene manipulation studies in which over-expression of clock gene in specific clock neurons can partially rescue behavioural rhythms and shortened free-running periods in old flies^9,10^.

There are two possible reasons for this result in overall reduction of clock-gene expression with age, a weakened signal from the central clock or the disruption of the peripheral oscillators. We found that robust molecular oscillations of PER persist in the dorsal neurons (DN_1–3_ and LN_d_) of the central clocks. This is consistent with a previous finding of robust PER cycling in s-LN_v_, DN_1_ and LN_d_^11^, but different to Umezaki’s 2012 study in which the amplitude of PER and TIM expression deteriorated with age in all central-clock cell groups (s-LN_v_, l-LN_v_, DN_1_, DN_2_ and LN_d_)^9^. Our finding supports the idea that a functional central oscillator is still intact in aged *Drosophila*. We infer that the effect of age is on the peripheral level rather than the central level. We showed reduction in PER and TIM expression without the total loss of rhythmicity in peripheral clocks throughout the fly bodies, which is consistent with previous findings in the fly heads^11,23,26^. It is clear that peripheral oscillators also dampen with age in mammals^27^, with reduced expression or weakened rhythmicity of several clock genes and/or their protein products in certain extra-SCN regions^7^. This can be explained by multi-oscillator theory and the impair of the coupling mechanism in old age^15,17^, leading to desynchrony among peripheral clocks.

Here, we report for the first time that a transient increase of both PER and TIM expression at the whole organism level occurs days before death. This signal was most obvious in TIM, and we infer that it can be used as a molecular marker of imminent death. The low signal background of *8.0-luc* makes it difficult to tell whether the aberrant increase of PER signal also existed in the central clock. However it has been reported that the central molecular clock in *Drosophila* remains efficient until the very last day of life^11^. In mammals, it has also been shown that the key function in the central clock is preserved^4,15^. Therefore the inference is that the effect is on the peripheral rather than the central level, which implies the loss of synchrony among peripheral clocks before death.

**Figure 8:**
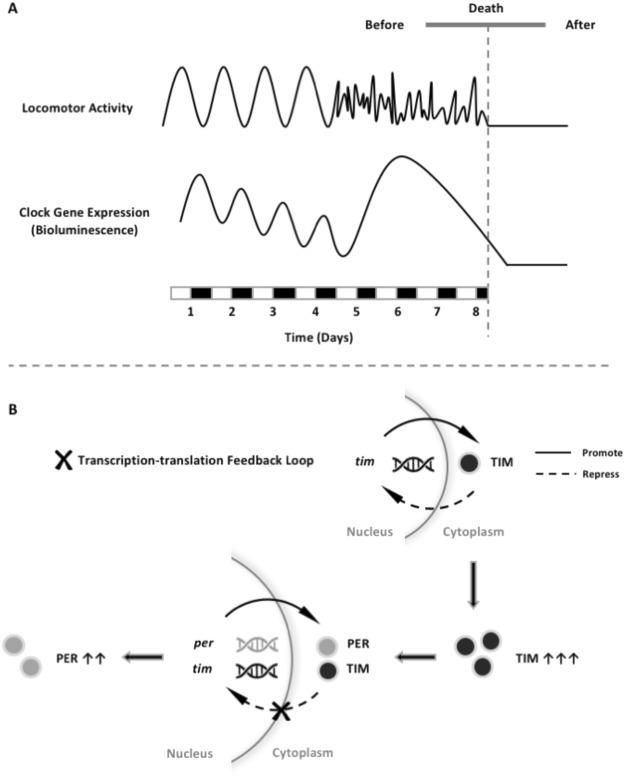
Schematic illustration showing the circadian clock disruption immediately preceding death at both the behavioural and molecular levels (**A**) and the hypothesised pathways responsible (**B**). The loss in the rhythmicity of locomotor activity in the last few days of life is mirrored by a simultaneous increase of clock-gene expression. The negative-regulated transcription-translation feedback loop is composed mainly of gene *per* and *tim*, their translational products PER and TIM in cytoplasm form a heterodimer which enters the nucleus to inhibit the transcription of *per* and *tim* themselves. The change of clock mechanism before death starts with the increasing transcription of *tim* without following a circadian pattern (here shown at day 5). We hypothesise this is due to the loss of regulation by the transcription-translation feedback loop. The increase of PER is likely to be a downstream effect of the aberrant expression of TIM. The changes of TIM may be related to its role in clock rhythmicity, cell cycle, DNA replication, DNA damage response, telomere length and integrity maintenance, and immune response.

The elevated signals occur in both clock-gene reporters *tim-luc* and *XLG-luc* with different effects on incidence, magnitude and phase (Fig. 6). We infer that this signal reflects the actual increase of clock-gene expression, rather than the changes in luciferase reaction which should affect both reporters equally. Furthermore, we hypothesize that the increase in TIM expression is the leading change, which in turn alters PER expression as a downstream effect. Prior to death, the increasing expressions of PER are still in phase with normal 24-hour LD cycles yet TIM expressions lose circadian pattern and distribute randomly. It implies that the change of clock mechanism before death is starting with the continuous increasing transcription of *tim* due to the loss of regulation by the transcription-translation feedback loop (Fig. 8). It is very likely that without the stabilization from TIM, PER in the cytoplasm cannot enter nucleus to inhibit its own transcription (negative feedback loop) thus resulting in an increase in expression. It also explains why some but not all of the flies (45.7%) show increase of PER expression, if TIM is around for PER at the right timing then PER expression is normal, otherwise PER expression increases.

Correspondingly, we hypothesize the aberrant increase of TIM immediately preceding death is central to the underlying mechanism of the final collapse of the circadian clock system. The loss in circadian behavioural rhythmicity (Fig. 7) can be explained by simultaneous increase of clock-gene expression. Such circadian clock changes (both gene expression and output behaviour) in the last days of life are not age-dependent because both young and aged flies shared similar pattern. The changes of TIM before death is related to its role as a clock gene essential in circadian rhythmicity, which is supported by the finding that *tim* null mutants show faster locomotor rhythm decline with age and shortened lifespan^8,28^.

Although the underlying mechanism is not fully revealed, *tim* must be important in the biology of aging and death beyond its roles in circadian rhythmicity. *Tim* functions in cell cycle progression, DNA replication, DNA damage response, and telomere length and integrity maintenance^29^, making it a multifaceted factor implicated in aging and death. *Tim* has drawn more and more attention in recent studies for its role in cancer^30–32^, also indicates its versatility. It also raises the possibility that immune response towards infection is one of the underlying reasons, given that the role of *tim* as a regulator of immune response has been recently revealed in Planarian^33^. A novel theory is also rising that through reprogramming of transcriptome in cells, aging turns off genes involved in homeostasis and turns on those involved in tissue-specific stresses, such as inflammation and DNA damage^34^. Furthermore, a recent study has reported that about 1% of the gene transcripts significantly increase in abundance post-mortem in zebrafish and mice, among which are genes involved in survival and stress compensation as well as developmental control and cancer^35^. Although the technique used limits the possibility to also measure the expression of these genes before death, it raises the possibility of a compensatory mechanism at the last stage of life. As a consequence, the outburst of *tim* expression in the days preceding death might reflect a stress, a compensatory, or an immune response.

We also investigated whether strong entrainment cues can improve the decline of molecular clocks in aging *Drosophila*. We used LD cycles and temperature-coupled LD cycles to try reversing the deterioration of the clock in peripheral oscillators. The impairment of the input pathway may be a possible explanation and is consistent with the finding that in these older flies the clock cannot be effectively entrained by light or temperature. A previous study has reported that LD cycles alone is not sufficient to rescue disturbance of locomotor activity in aging flies, but the use of LD (12:12) coupling temperature cycles (25 °C/21 °C) turned out to be effective^11^. Our finding suggests a clock-independent temperature effect may be involved. Lower temperatures (such as 21 °C) prolong longevity in *Drosophila* and slow down the age-associated sleep decline, likely through non-circadian mechanisms^8,36^.

Circadian coordination has a pronounced impact on physiological functions, overall health, and disease susceptibility^37^, it is reasonable to infer how lifespan and health would suffer when the circadian system is challenged. Therefore robust and entrained circadian rhythms could potentially lead to better health and increased longevity^7^. However aiming at entraining the central clock alone is not efficient enough as we find that the peripheral clocks are more seriously affected by aging. A number of studies have shown that regular routines of mealtime and exercise improve circadian consolidation in elderly individuals, addition with light routines, these timing cues might help substitute for the loss of internal desynchrony^5^. We also report a remarkable disruption of the circadian clock during the last days of life at both the molecular (Fig. 6) and behavioural levels (Fig. 7 and diagrammatic illustration Fig. 8). Our finding indicates not only the underlying cause of the circadian clock disruption before death, but also the exciting potential of *tim* to be a marker of imminent death, a link for further mechanism exploration and even a basis for intervention.

## Methods

### Fly stock

*Drosophila* transgenic reporter lines *8.0-luc*, *XLG-luc* and *tim-luc*, and wild type flies were reared on growth-media (Carolina, USA) loaded in standard plastic vials. Flies were kept at 12:12 LD cycles and at temperature 25 °C, and transferred to fresh vials once a week. Flies were collected within 2 days of eclosion and survival of mated male flies in vials were monitored until their death, showing average longevity of 50–70 days. Young (10-day-old), middle-aged (30-day-old) and old (50-day-old) mated male flies were used. All flies of different ages were kept under identical circumstances.

### Measurement of clock-gene expression

Transgenic flies for real-time bioluminescence recording of clock gene *per* and *tim* expression were used in this study, as described previously^38^. Individual flies were transferred into a single well of a white opaque 96-well microplates (Greiner Bio-one). Plates were loaded into a Multimode Plate Reader (EnSpire, PerkinElmer) kept inside an incubator (Percival Scientific Inc, USA). Experiments were conducted in DD, LD cycles (12:12) at constant temperature (25 °C), and LD cycles (12:12) coupled with temperature cycles (light on at 25 °C and light off at 21 °C).

### Monitoring of locomotor activity

Locomotor activity of wild type flies was monitored automatically using Trikinetics *Drosophila* Activity Monitors (Waltham, MA, USA), as previously described^39^. Individual flies with known age (10-day-old, 30-day-old and 50-day-old) were transferred into glass tubes (8 cm long and 5 mm diameter) with grow media at one end, sealed with plastic plugs, and plugged with cotton at the other end. Locomotor activity, temperature and relative humidity were recorded every five minutes for an entraining period of 13 days in LD and then in DD until most flies died.

### Statistical analysis

Data are shown in mean and standard error (Mean ± SEM). Statistical significance of the amplitude and acrophase of time series data among young, middle-aged and old transgenic flies were determined using two-way ANOVA with Bonferonni’s post hoc test^23^. ClockLab (Actimetrics, USA) was used for the analysis of periodicity and rhythmicity in locomotor activity of wild type flies. Periodicity was tested by Chi-square periodogram and rhythm strength was measured by FFT values^8^.
